# Determinants of Genetic Structure in a Nonequilibrium Metapopulation of the Plant *Silene latifolia*

**DOI:** 10.1371/journal.pone.0104575

**Published:** 2014-09-08

**Authors:** Peter D. Fields, Douglas R. Taylor

**Affiliations:** Department of Biology, University of Virginia, Charlottesville, Virginia, United States of America; University of Umeå, Sweden

## Abstract

Population genetic differentiation will be influenced by the demographic history of populations, opportunities for migration among neighboring demes and founder effects associated with repeated extinction and recolonization. In natural populations, these factors are expected to interact with each other and their magnitudes will vary depending on the spatial distribution and age structure of local demes. Although each of these effects has been individually identified as important in structuring genetic variance, their relative magnitude is seldom estimated in nature. We conducted a population genetic analysis in a metapopulation of the angiosperm, *Silene latifolia*, from which we had more than 20 years of data on the spatial distribution, demographic history, and extinction and colonization of demes. We used hierarchical Bayesian methods to disentangle which features of the populations contributed to among population variation in allele frequencies, including the magnitude and direction of their effects. We show that population age, long-term size and degree of connectivity all combine to affect the distribution of genetic variance; small, recently-founded, isolated populations contributed most to increase *F*_ST_ in the metapopulation. However, the effects of population size and population age are best understood as being modulated through the effects of connectivity to other extant populations, i.e. *F*_ST_ diminishes as populations age, but at a rate that depends how isolated the population is. These spatial and temporal correlates of population structure give insight into how migration, founder effect and within-deme genetic drift have combined to enhance and restrict genetic divergence in a natural metapopulation.

## Introduction

Sewall Wright [1] introduced the study of geographic population structure and its genetic consequences with the Island Model. In the Island Model, populations are assumed to be stable and interconnected by migration, with population differentiation (*F*_ST_) at neutral loci generated by drift and diminished by gene flow among populations. When the simplifying assumptions of the island model are violated, a number of additional factors are necessary to understand population genetic differentiation [2], [3]. In metapopulations, where demes experience extinction and recolonization, there are broad conditions under which the founder effect can be a powerful structuring mechanism [4], and population differentiation can occur despite high levels of gene flow. The importance of founder effects depends on several parameters including migration, extinction and colonization rates, the number of founding propagules, as well as the fraction of demes from which the colonists originate [2], [3].

Empirical evidence that founder effects can drive population differentiation has been gathered from age-structured populations. For example, elevated population structure among newly established demes in metapopulations is consistent with founder effects generating increased population structure, with subsequent gene flow reducing genetic differentiation among populations as they age [5]–[9]. Although these classic studies demonstrate that metapopulation processes (extinction and recolonization) can influence genetic differentiation, it is important to estimate their importance relative to other structuring mechanisms such as local adaptation, which will also contributed to increased population genetic structure [10], [11].

Recent advances in evaluating the causes and consequences of population structure have benefitted from the development of a Bayesian model-based approach known as the *F*-model [12]–[17]. This approach has an advantage over previous methods in that rather than estimating “global” values of genetic differentiation, it takes into account the biological reality that local populations differ in their effective sizes and migration rates and, therefore, their degree of differentiation from other populations [12]. Foll and Gaggiotti [18] introduced a hierarchical formulation of the *F*-model that uses population-specific measurements to obtain priors for *F*_ST_, then estimates the proportional contribution of population-specific variables generating among population variance in allele frequencies.

We used the hierarchical Bayesian approach of Foll and Gaggiotti [18] to understand variation the causes *F*_ST_ in a well studied plant metapopulation. Our objective was to estimate how population structure results from equilibrium process such as those in the island model (e.g. drift, migration) versus non-equilibrium factors that operate in metapopulations (e.g. extinction and recolonization with founder effects). To accomplish this, we applied the *F*-model formulation to a long-term data set of the plant, *Silene latifolia*, where the size and spatial distribution of demes was known, and could be combined with information about their demographic history and age structure. We used fine-scale sampling, microsatellite genotyping, and the application of an *F*-model based approach to explore the spatial and temporal features of populations that affect their contributions to the observed distribution of *F*_ST_, including simultaneous estimates of the magnitude and direction of these effects.

## Materials and Methods

### Study Organism

*Silene latifolia* Poir. ( =  *S. alba*, Caryophyllaceae) is a short-lived perennial plant that is broadly used as a model system for studying sex determination, sex chromosome evolution, host-pathogen dynamics, biological invasions, organelle evolution, sexual dimorphism, sex ratio evolution, and evolution in structured populations [19].

**Figure 1:**
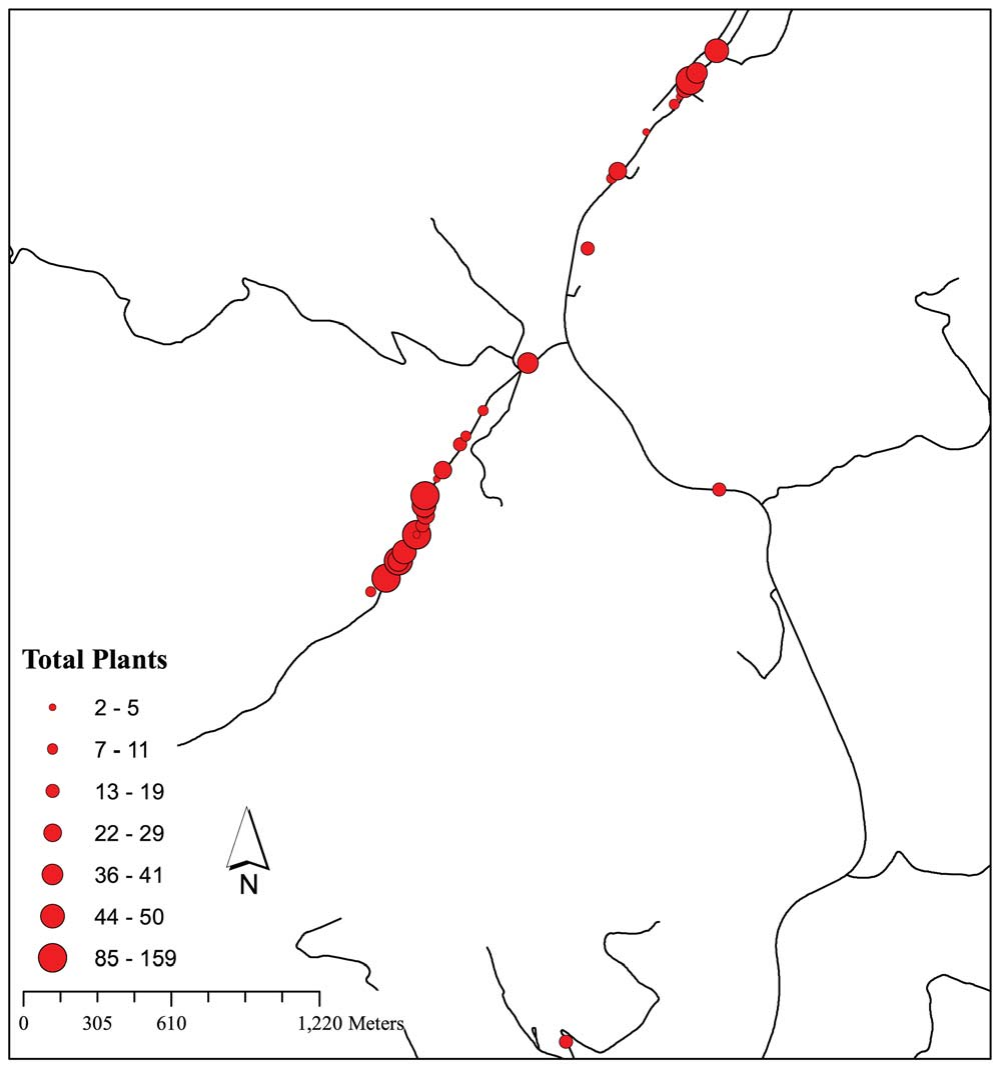
Map of the focal populations of the *S. latifolia* metapopulation located in Giles and Craig County, VA, USA sampled in the presented analysis. Circles represent individual populations, where the size of the circle indicates the total number of plants located within our grid. Black lines represent both the assumed grid of population arrangement and small country roads. Due to the topology of the focal area (mountain, valley systems), it was assumed that pollinators move along the linear grid, rather than crossing over ridges.

We studied a *S. latifolia* metapopulation located in Giles and Craig Counties, Virginia, USA (Figure 1). This region has been the subject of a 20+ year study of population dynamics and genetic structure in more than 800 populations [5], [20]–[25]. An annual census of an approximately 25×25-km area adjacent to Mountain Lake Biological Station has been conducted since 1988, recording the location and sex of *S. latifolia* individuals along ∼150 km of predominantly roadside habitat. The structure of the data and how it was collected is reported in Antonovics *et al.* [20]. Briefly, the roadside habitat is divided up into ∼40 m grid units which we classify as populations. Previous studies have indicated that the utilized grid system may encompass one or two genetic neighborhoods on average [26], [27], levels of extinction and colonization scale consistently with grid segment size [20], [28], and that dispersal distances are such that populations separated by 20 meters were almost panmictic, while populations separated by 80 meters were nearly isolated [24]. Dispersal of pollen is insect mediated, primarily noctuid moths, and seed dispersal is passive and happens at a much smaller scale [22]. Much longer distance dispersal is likely mediated by human activity [29], with road grading and mowing as possible mechanisms [24]. Which units are occupied and the number of plants in each occupied population are recorded annually. Important phase transitions, such as extinction and colonization events, are confirmed with a second census during the same season. Time since colonization (or population age) is based on the year plants were first observed in a given grid unit. We identified extinction as the disappearance of plants from a grid unit for a single year. A previous study has indicated that seeds can remain viable in the soil for approximately four years, raising the possibility of a seed bank [30]. For our purposes, we treat the recolonization of a population by propagules in the seed bank as roughly equivalent as recolonization by propagules from a nearby site. The census data provide the demographic data, spatial relationships among populations, and extinction/recolonization dynamics used in this study (Table 1).

**Table 1:** *S. latifolia* populations used in the genetic analysis.

	*F*_ST_ Statistics			Factors				
Population ID	Sample Size	Mean	Mode	95% HPDI	Age	Population Size	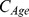	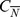
Population 1	24	0.183	0.174	[0.128; 0.248]	21	22.038	2.15	44.543
Population 2	12	0.2	0.188	[0.111; 0.302]	3	1.246	0.143	1.246
Population 3	7	0.108	0.096	[0.041; 0.186]	3	4.485	0.51	9.304
Population 4	5	0.177	0.147	[0.0719; 0.296]	1	4.82	0.228	9.675
Population 5	19	0.064	0.058	[0.0298; 0.1]	21	13.477	6.192	54.116
Population 6	50	0.046	0.043	[0.0272; 0.0665]	21	9.023	4.37	18.625
Population 7	22	0.056	0.051	[0.0247; 0.0886]	18	5.956	5.308	23.917
Population 8	24	0.048	0.045	[0.0244; 0.0744]	21	10.309	5.218	21.658
Population 9	39	0.041	0.039	[0.0242; 0.06]	20	9.389	6.54	38.816
Population 10	21	0.044	0.039	[0.0197; 0.0718]	12	3.774	3.924	15.602
Population 11	18	0.043	0.038	[0.0199; 0.0715]	21	8.873	6.098	27.936
Population 12	47	0.048	0.045	[0.028; 0.0713]	18	1.733	5.362	7.004
Population 13	8	0.071	0.054	[0.014; 0.142]	21	7.827	6.256	31.624
Population 14	20	0.064	0.059	[0.0287; 0.105]	17	4.16	3.808	9.361
Population 15	44	0.024	0.022	[0.011; 0.0376]	10	6.521	1.663	8.719
Population 16	11	0.156	0.139	[0.0743; 0.248]	21	5.255	4.213	13.407
Population 17	8	0.099	0.088	[0.037; 0.165]	12	2.051	2.627	6.986
Population 18	19	0.139	0.129	[0.0784; 0.209]	4	3.84	0.876	13.077
Population 19	10	0.179	0.166	[0.0888; 0.275]	3	1.503	0.474	3.963
Population 20	16	0.106	0.098	[0.0533; 0.163]	1	1.392	0.139	3.147
Population 21	10	0.166	0.15	[0.0809; 0.269]	1	1.507	0.055	1.543
Population 22	6	0.155	0.137	[0.0669; 0.252]	12	5.771	1.175	11.573
Population 23	36	0.11	0.106	[0.0724; 0.151]	11	1.807	1.077	3.622
Population 24	27	0.156	0.148	[0.0962; 0.223]	3	2.345	0.19	2.408
Population 25	8	0.218	0.205	[0.119; 0.334]	1	1.486	0.066	1.506
Population 26	21	0.116	0.107	[0.0619; 0.173]	20	4.22	1.343	5.159
Population 27	8	0.124	0.111	[0.0572; 0.2]	21	7.679	2.696	15.767
Population 28	4	0.075	0.066	[0.0205; 0.139]	21	17.038	2.358	21.839
Population 29	15	0.124	0.113	[0.0649; 0.188]	21	19.11	2.283	21.082
Population 30	44	0.113	0.107	[0.0718; 0.161]	21	45.22	2.335	51.538
Population 31	24	0.143	0.133	[0.0885; 0.209]	4	1.535	0.614	3.454
Population 32	29	0.136	0.129	[0.0818; 0.193]	21	26.265	2.568	39.887
Population 33	44	0.104	0.101	[0.0677; 0.143]	1	1.161	0.128	2.166

Population ID  =  census ID of individual populations, Mean, Mode, and 95% HPDI of the RJ-MCMC derived posterior model estimates, Age  =  number of continuously occupied years of individual populations, Population Size  =  harmonic mean population size of each sampled population, 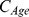 =  composite character composed of the sum distance between a focal population to all other extant populations and a focal populations age, and 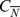  =  composite character composed of the sum distance between a focal population to all other extant populations and a focal population's size.

### Spatiotemporal Data

We estimated characteristics of populations that are thought to significantly influence the magnitude of genetic differentiation among demes in a metapopulation, including population size, population connectivity (a characteristic that will influence gene flow between populations), and population age (a factor that estimates the when founder effects occurred and opportunities for subsequent migration).

Given our incomplete knowledge of the contribution and life-span of an individual population's seed bank, we estimated harmonic mean population size (hereafter population size) based upon the number of plants (both flowering and vegetative) occupying a given grid-unit throughout the time of the annual census as: 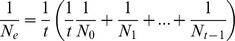



Population size estimates derive from the occupancy of a given site throughout the full census period. Detailed surveys on a subset of the metapopulation have been conducted to estimate the extent that our non-invasive census protocol sacrifices precision and accuracy. In the survey, we carefully searched the vegetation and when plants were found, the shoots were carefully traced down to the ground to distinguish individuals that were rooted close together. Non-flowering plants were also counted. Population sizes in the detailed survey were highly correlated with population size from the metapopulation census as assessed with a Pearson Product Moment Correlation (0.74, P<0.001). Population size in the metapopulation census (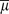  = 14.32) is lower than in the detailed survey (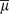  = 36.91) because clustered plants many be counted as single individuals in the annual census. The census, therefore, accurately measures relative population sizes, but likely underestimates absolute size.

To estimate population age, we assumed sites unoccupied for a single year went extinct, and that plants occupying that site in the future were classified as colonizations. This reflects the conservative assumption that recolonization from neighboring sites and recolonization from the seed bank involve similar bottlenecks of genetic diversity; if this assumption were violated, and new colonization from a seed bank did not involve the same degree of bottleneck, this would tend to obscure any differences we observed between younger and older populations. Following extinction and re-colonization, age is calculated as the number of years a site has been occupied up until the time of collection. Given this operational definition of population age, individual sites could vary in age from one to 21 years (the extent of the demographic census started in 1988). Populations in the age class of 21 years might reasonably be considered as a heterogeneous grouping of extant populations given that some populations may actually be older than the extent of the current long-term census.

There are a number of methods described in the literature that have been used to estimate population connectivity. The majority of ecological studies have used a nearest neighbor/patch approach, or distance to multiple neighbors within a limited neighborhood of a focal patch (or buffer) [31]. However, these measures have been shown to be poor predictors of ecologically important metapopulation dynamics such as colonization potential. To measure population connectivity, we generated two composite variables, each of which includes a negative exponential dispersal kernel and accounts for distance to all other potential extant populations for gene flow [32]. Each variable has the following structure:
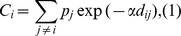
where 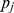 is in 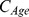 the proportion of the censuses for which a given site was occupied (1/21 – 1) or in 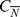, the population size over the life span of the population, of some population *j*; 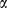 is a parameter scaling the effect of distance on dispersal, and 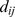 is the road network based distance between a target population *i* and source population *j* [31], [32]. Pair-wise distances were calculated using a network constructed based upon the public roadway system, using ArcGIS (ESRI) Network Analyst tool. Given the mountain-valley geographic topology of the area, this network-based approach is more appropriate than standard Euclidean distances. 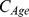 or 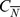 are indicative of a population's probability of receiving migrants, whether through seeds or pollen, though these are modulated by the opportunity for gene flow over-time or through apparency to pollinators, respectively.

### Sampling

We focused on one of the nine metapopulation sections, which are classified with an *ad hoc* partitioning of the mountain-valley system topology of the area, and is consistent with the expectation that each are mostly genetically isolated [27]. We sampled plants from 33 spatially distinct populations during peak flowering in the summer of 2008. Sites occupied by < 4 plants were excluded since we could not reliably estimate gene frequencies in our estimation of *F*_ST_. *S. latifolia* is identified as in introduced weed in the study area, and does not require specific permits to collect tissue samples. We collected leaf tissue from every plant in the population, or up to 50 individuals in the larger populations, and stored the leaves with silica gel (Sigma, USA).

Genomic DNA was extracted and amplified following established microsatellite techniques for *S. latifolia* [33]. We genotyped each plant at 8 microsatellite loci. Microsatellites were derived from multiple sources [34]–[36]. PCR amplification was conducted using previously published methods [37]. Briefly, PCR products were amplified with the forward primer end-labeled with a fluorescent dye, either 5(or 6)-FAM, NED, TAMRA, JOE, or VIC. Three to four PCR products of different loci were then pooled together and added to a loading buffer containing formamide and GENESCAN 400HD ROX size standard (Applied Biosystems, USA). Following five minutes of denaturing at 95°C, fluorescently labeled fragments were separated on an Applied Biosystems 3130 sequencer and analyzed with GENEMAPPER v3.0 software (Applied Biosystems, USA). Allele binning was accomplished using the software TANDEM [38].

### Statistical Methods

Overall, the data involved 730 plants each with a multi-locus genotype derived from eight microsatellite markers. The plants were associated with one of 33 populations that had size, age and connectivity data collected from the long-term demographic dataset.

We used the program GenoDive [39] to estimate global summary statistics of population structure for our molecular markers, including observed (*H*_O_) and expected (*H*_E_) heterozygosity, *F*_ST,_ and Jost's *D* [40]. We used the the analytical software R [41] and the package hierfstat v. 0.04-10 [42] to calculate rarefied allelic richness. We used the hierarchical Bayesian method of Foll and Gaggiotti [18], implemented in the program GESTE v. 2, to evaluate the effect of spatial and temporal characteristics of populations on the magnitude of genetic differentiation among populations. The method implemented in GESTE estimates *F*_ST_ values for each sampled population using the approach first proposed by [14] and relates them to environmental factors using a generalized linear model framework [43]. Specifically, 
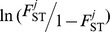
 is related to models of the form 
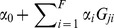
, where 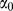
is a constant, 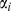
is a regression coefficient of the effect of factor 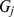
from some pop *j* affecting the response variable, in this case individual population *F*_ST_ values. We considered four factors, thus generating 16 alternative regression models The simplest null model included only a constant term, whereas the most complex model included a constant and all four factors. Because the two connectivity variables, 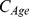
and 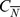, were found to be significantly correlated with a Pearson product-moment correlation coefficient of 0.6141 (95% confidence interval (CI) 0.3432–0.797; P = 0.0001), we additionally ran GESTE excluding one or the other connectivity measure. GESTE does not allow for testing a full model that would include all four pairwise interactions. The method provides posterior probabilities for the alternative models using a RJ-MCMC approach [18]. Under this framework, the model with the highest posterior probability is the one that best explains the data [18], [43]. We followed the method of Gaggiotti *et al.* [43], using 10 pilot runs of 1000 iterations to obtain parameters of the proposal distributions used by the MCMC, followed by an additional burn-in of 5×10^6^ iterations and a thinning interval of 50, with a final iteration sample size of 60,000 on which the model fit probability was based. Convergence was assessed visually using the Plot GESTE.exe function included in the GESTE v.2 distribution. Using this method, we identified the model that best explained the observed genetic structuring. The magnitude and direction of a spatiotemporal character on genetic structuring was inferred from estimates of the regression coefficients from the model with the highest posterior probability.

## Results

Global summary statistics (Table 2) revealed a high degree of population structure, with an overall *F*_ST_ = 0.103, and a range among markers of 0.055 to 0.231. This was similar to previous estimates in this metapopulation using allozymes [0.134, 21]. The observed levels of population genetic structure are also consistent with other published datasets on plant metapopulations using microsatellite markers, including other *Silene* species. Previous research has shown that the sub-structuring of alleles is not the consequence of PCR artifacts such as null alleles [34]–[36]. We estimated a significantly negative Pearson product-moment correlation coefficient between the genetic diversity of individual populations and that population's contribution to *F*_ST_ (r = −0.3601, 95% CI −0.6260, −0.0191; P = 0.0396; Table S1). *D* had a mean of 0.131, and a range of 0.047–0.337.

**Table 2:** Global estimates of genetic diversity and variation in allele frequencies.

Locus	*N*	*H_o_*	*H*_s_	*D*	*F_ST_*
*slat_18* ^1^	10	0.338	0.592	0.158	0.09
*slat_32* ^1^	9	0.303	0.673	0.337	0.135
*slat_33* ^1^	3	0.07	0.13	0.051	0.231
*slat_48* ^1^	2	0.078	0.202	0.047	0.143
*slat_72* ^1^	12	0.325	0.593	0.103	0.055
*slat_85* ^1^	16	0.324	0.538	0.178	0.125
*SL_8^2^*	43	0.693	0.819	0.245	0.051
*SV_11* ^3^	11	0.305	0.573	0.19	0.122
***Overall***	**13.25**	**0.304**	**0.515**	**0.131**	**0.103**

Variables are the number of alleles (*N*), observed heterozygosity (*H_O_*), Expected heterozygosity (*H_S_*), Jost's *D* (*D*) and global variation in allele frequencies (*F*_ST_). Overall, populations showed a high degree of substructure, as has been observed in other *Silene* metapopulations. Population structure was high for each marker. Our lowest *F*_ST_ corresponded to the only marker composed of a dinucleotide repeat. ^1^ [34], ^2^ [35], ^3^ [36].

The magnitude and direction of the effect of each of the spatiotemporal characters on genetic differentiation was inferred from the estimates of the regression coefficients for the most probable model. The four parameters affected genetic differentiation in directions that were consistent with both theoretical expectations concerning the role of spatial-temporal dynamics affecting the distribution of *F*_ST_ across a metapopulation [8], as well previous research on the system (Table 3, 4; Fig. S1–S3). For example, recent founder effects and small population size enhanced *F*_ST_, as was previously described theoretically by Whitlock [8], and has been shown empirically in a number of other systems across a broad range of taxa known to occur as a metapopulations [6], [9] [5], [6], [9], including the focal plant metapopulation. However, the parameters of population age and population size were not individually components of models with the highest posterior probabilities. Instead, a composite parameter that combined population age and the degree of connectivity, 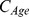, provided the highest posterior probability (0.752) model (Table 3, 4; Fig. S2), consistent previous studies by Moilanen and Nieminen [31] for predicting extinction and colonization processes. To identify which population-level character contributed the most to global *F*_ST_, we summed the posterior probabilities of models that included individual factors. Among these, population age (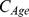, with a posterior probability of 0.994), emerged as the one factor that had an overwhelming effect on a population's contribution to the global value of *F*_ST_ (Table 3). The 14 other models attained <0.1 posterior model probability.

**Table 3:** Sum of posterior probabilities of models that include a given factor.

Factor	Sum of the posterior probabilities
Population Age	0.0935
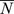	0.0541
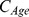	**0.994**
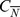	0.124

Bold value indicates factor with highest score.

**Table 4:** Posterior probabilities of all 16 models.

Model	Pr	Factors included
**5**	**0.75**	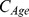
13	0.10	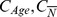
6	0.08	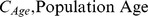
7	0.04	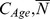
15	0.01	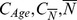
14	0.01	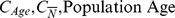
1	0.00	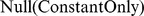
8	0.00	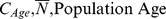
16	0.00	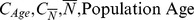
2	0.00	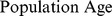
9	0.00	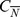
3	0.00	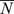
10	0.00	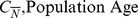
4	0.00	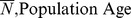
11	0.00	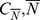
12	0.00	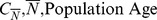

Our most probable model (bolded) included the composite variable of population age and population connectivity. The second most probable model includes composite variables of population age and connectivity, and population size and connectivity.

Comparison of the GESTE derived estimates of individual factors contribution to variation in *F*_ST_ across individual models, i.e. all models including a given factor, showed that population age was the most important determinant of population genetic structure, followed by population size, and that each of these were influenced by the degree of connectivity. GESTE analyses run excluding population size, 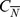, resulted in the 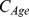 factor remaining the dominant factor increasing *F*_ST_ (Table S2.a). The reverse analysis, which excluded 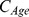 and included 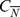, resulted in a model with a low posterior probability (Table S2.b)

Posterior estimates of the regression parameters of the model that only included Constant + 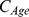
were significantly negative (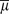 = −0.439, mode  = −0.447, 95% HPDI [−0.680; −0.200]), indicating that the initial increase in *F*_ST_ resulting from founder effects is reduced over time, presumably by subsequent migration (Table 5; Fig. S3). Posterior estimates of the regression parameters of the next best model indicated a consistent effect of population age, 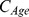 (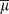 = −0.575, mode  = −0.600, 95% HPDI [−0.857; −0.265]), and a positive effect of populations size, 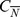 (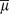  = 0.207, mode  = 0.201, 95% HPDI [−0.079; 0.472]) indicating that younger and smaller populations contributed proportionately greater to the overall magnitude of genetic differentiation, and that proportional effects were modulated through population connectivity. Regression parameters for models excluding one or the other connectivity factor were of similar direction and magnitude (Table S3a, S3b).

**Table 5:** Posterior estimates of regression parameters for the model with the highest posterior probability.

Regression coefficient	Factor	Mean	Mode	95% HPDI
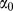	Constant	−2.26	−2.25	[−2.50; −2.00]
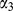	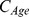	−0.435	−0.445	[−0.687; −0.193]
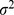	-	0.369	0.316	[0.168; 0.617]

Parameter estimates are consistent with theoretical expectations (e.g. older and larger populations contribute proportionately less to the global *F*_ST_).

## Discussion

In the present study, we simultaneously identified several important spatiotemporal parameters that contributed to population genetic differentiation. While previous research on other plant metapopulations has shown that founder effects contribute to population differentiation [5]–[7], [21], [22], [44]–[46], none of these previous studies have been capable of quantifying the relative magnitudes of these effects.

**Figure 2:**
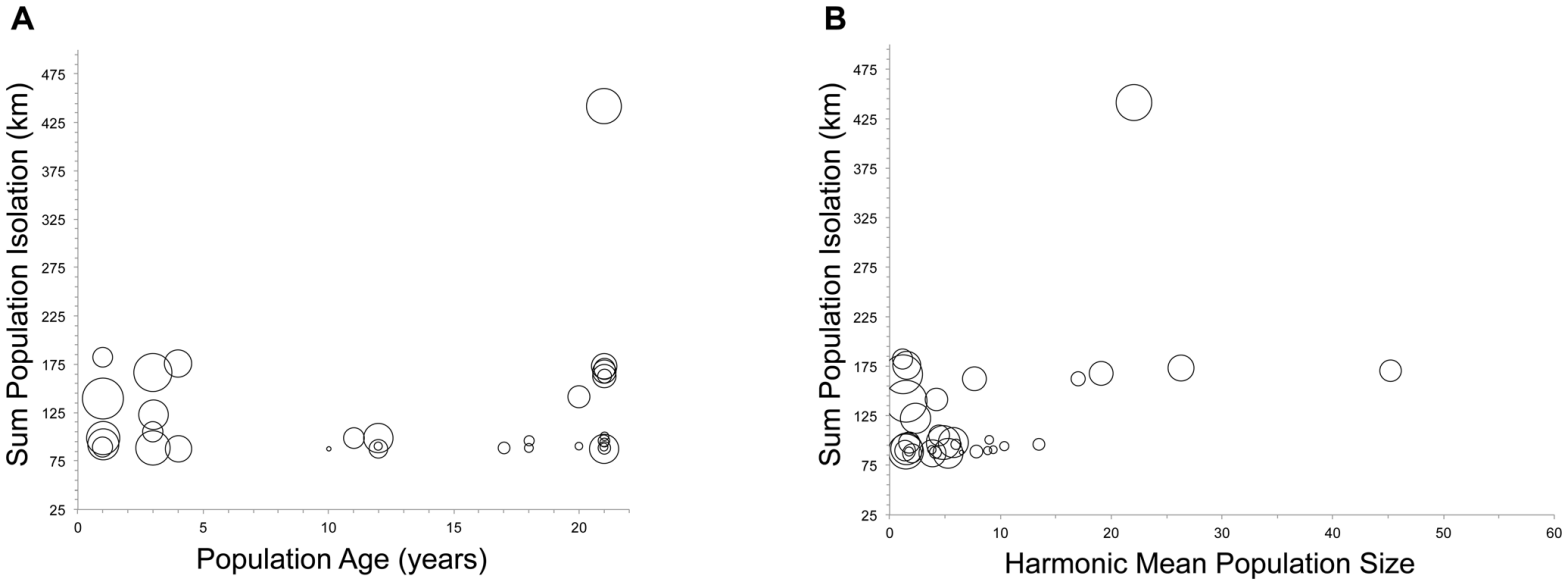
The effect of population age, connectivity, and population size on *F*_ST_. Individual population *F*_ST_ is represented by the size of the circle, where larger circles represent larger *F*_ST._ (A) Simultaneous effect of population connectivity and population age effects on *F*ST, and (B) of population connectivity and population size on *F*_ST_.

In the *Silene latifolia* metapopulation, population size, degree of connectivity and population age were each important determinants of population structure, but composite characters (so-called connectivity scores; [31]) were most important. This implicates the extinction and recolonization of demes, followed by subsequent opportunities for gene flow as a population ages, as the most important driving force in the genetic differentiation among populations in this system. This point is particularly clear when observing Population 1 which is part of the oldest age class but also maintains one of the higher *F*_ST_ values due to isolation (Figure 2; upper right quadrant). The genetic consequences of founder effects and within-population genetic drift, as modulated by long-term population size, were influenced by the degree of population connectivity and hence the opportunities for gene flow.

The *S. latifolia* metapopulation is neither a true Island Model, nor an idealized metapopulation [47]; populations are characterized by frequent colonizations and extinctions, on the order of 5-20% per year [20], [27], [48], [49], but populations vary in size, dispersal is limited, and within population dynamics are important relative to the time scale of the study. Colonizations are likely to be source-size and distance dependent, as is typical in other metapopulation systems [50]–[52], though this point has not been explicitly tested. Our findings support the general notion that founder effects during colonization can enhance genetic differentiation among populations [5], but we additionally show that the magnitude of these effects are large (Figure 2) relative to the structuring mechanism assumed in most models, i.e. genetic drift among extant demes as described by Wright's Island Model [1].

It is a nearly universal observation that a given species range will be composed of populations that are patchily distributed in space. There are clear similarities in the extinction/colonization and incidence parameters in *Silene* versus other model systems; e.g. the Glanville fritillary (*Melitea cinxia*) metapopulation in Finland [53]. Spatial patterns of colonization and extinction responded similarly to scaling in regional studies of sunflowers in the mid-west (*Helianthus annuus*; [27])(Helianthus annuus; [28]). Metapopulation dynamics have also been documented in the fresh water crustacean *Daphnia longispina* and *D. magna* (Cladocera), where the founding of new populations by single or few individuals was followed by predominantly clonal reproduction. This lead to an initially large increase in *F*_ST_ in newly founded populations followed by the gradual dissolution of variation in allele frequencies through limited gene flow [9]. While the direct quantitative values will obviously vary from system to system, one might expect that non-equilibrium dynamics that result from the extinction and recolonization of local demes would be most important in systems like these, where there is a high turnover of demes. On the other hand, drift can be expected to be a less powerful structuring mechanism when demes are larger and more permanent. For example, Cosentino [32] show that connectivity and wetland area were the most important factors driving variation in allele frequencies amongst populations of the tiger salamander *Ambystoma tigrinum*.

The observed global *F*_ST_ in the present study was lower than has been previously reported. Specifically, McCauley [21], [44] reported mean estimates *F*_ST_ = 0.134 in seven allozyme markers. Previous research has suggested that the *F*_ST_ of microsatellite markers will be smaller than for allozymes due to the higher heterozygosity of microsatellite markers and the inverse relationship of *F*_ST_ and marker heterozygosity [54]. In addition, the estimated *F*_ST_ in the present study is likely an underestimate due to our sampling scheme, which include almost all individuals rather than a small sample. Specifically, Whitlock [8] describes the sampling scheme used on a metapopulation of the forked fungus beetle (*Bolitotherus cornutus*) as encompassing almost the entirety of the population. In such cases Whitlock [8] suggests the use of a hypergeometric sampling correction to account for the sampling error normally assumed by a binomial sampling error. Recently Wood *et al.* [55] describe a more recent analysis of the same focal *Bolitotherus cornutus* metapopulation, though not exactly the same section as Whitlock [8]. Wood *et al.* [55] report much lower values of *F*_ST_ values for microsatellite markers, which might be similarly affected by the issues described by Edelaar *et al.* [54] wherein markers showing larger numbers of alleles, e.g. microsatellites, will show a general trend towards smaller values of *F*_ST_ compared to markers that have fewer alleles (allozymes), but also because a multinomial-hypergeometric correction was not included for the estimation of *F*_ST_.

Theoretical explorations of metapopulation dynamics have shown that spatiotemporal characters should have direct effects on each other, and we found empirical evidence for this. For example, we found a significant positive pairwise correlation (Pearson Product Moment Correlation  =  0.478, P<0.01) between population size and population age; older populations also tended to be larger. Thus, while we show that both of these effects are potentially important in generating genetic divergence, our ability to fully disentangle the effects of age and population size is limited. Neither population size nor age was significantly correlated with population connectivity (P>0.05).

While the present approach provides insights into the biology of the *Silene* metapopulation, additional insights would be gained from sampling populations over time. Lamy *et al.* [56], longitudinally sampled populations of the freshwater snail, *Drepanotrema depressissimum,* to show that demographic survey-derived assessments of extinction were incorrect; rather than a true metapopulation, they detected persistent demes with genetic diversity driven by different population sizes and rates of immigration. Similarly, Robinson *et al.* [57] applied a novel use of approximate Bayesian computation (ABC; [58]) to genetic data from a *Daphnia magna* metapopulation described by Haag *et al.* [9]. They concluded that some populations act as persistent sources, similar to a continent-island model.

Discernment of presence/absence of extant populations within a census is highly efficient in *S. latifolia* (see above). However, previous research has also suggested the presence of a seed bank [30], though allele frequencies observed in the seed bank are highly consistent with above ground cohorts [30]. We believe our *ad hoc* measure of population extinction is consistent with the theoretical expectations of the effect of founder effects on the distribution of variation in allele frequencies [8]. Temporally spaced samples as in Lamy *et al.* [56] could more conclusively confirm this particular hypothesis, as well analyses such as the one presented by Robinson *et al.* [57]. Further investigations such as those carried out by Lamy *et al.* [59] and Fréville *et al.* [60], where the expectation of a seed bank/resting form is assumed as part of larger model of demographic dynamics, will allow for a better understanding misidentification of extinctions would effect our inference of metapopulation dynamics.

Thus far, we have focused on evolutionary processes that are driven by non-selective, drift related processes. However, previous studies have indicated the potential for metapopulation structure to have a significant outcome on selective dynamics and vice versa [61]. Since we derived our molecular markers from non-coding regions, they were assumed to be neutral and to be unlinked to functionally important genomic regions [34]–[36]. Although our panel of markers does not exhibit discontinuities in their distribution characteristic of outliers, given our limited panel size and the complexity of detecting outliers in hierarchically structured populations [62], we have only limited power to test this assumption.

Natural selection could have a powerful effect on population structure, even when the alleles under selection are not closely linked to marker loci. Studies in this *S. latifolia* metapopulation have confirmed that population genetic structure, where individuals in closer proximity tend to be more related than expected from chance, can have a significant negative average effect on individual fitness through the expression of deleterious recessive alleles [25]. Because inbreeding depression will reduce average fitness and therefore population size, drift processes could be enhanced through selective reductions in population size. Because many of the young, recently colonized populations will experience inbreeding depression, gene flow from other populations may be enhanced beyond neutral expectations due to heterosis [63], [64]. This initial enhancement in gene flow could, combined with stochastic dynamics associated with non-equilibrium metapopulation conditions, enhance the observed reductions in population specific *F*_ST_ over time. Thus, our analyses are unable to disentangle neutral and the selective effects of spatiotemporal metapopulation structure in generating variation in allele frequencies among populations.

The present study demonstrates that the appropriate combination of long term ecological data and population genetic analyses may be a powerful tool for studying the mechanisms that generate population structure. Spatiotemporal characters of populations have long been hypothesized to have significant effects in generating population genetic differentiation, and its selective consequences. This fact seems to be particularly true in the *S. latifolia* metapopulation, where population connectivity, age and population size all combine to drive population genetic differentiation.

## Supporting Information

Figure S1 **Posterior 95% HPDI estimates of individual population's** ***F*** **-model based estimate of** ***F*** **_ST._**
(TIFF)

Figure S2 **Posterior model probabilities for GESTE run including all four factors.**
(TIFF)

Figure S3
**Plots of the GESTE estimated ln(** ***F*** **_ST_/1-** ***F*** **_ST_) against (A) population age, (B) Population Size, (C)** 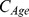 **, and (D)** 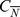 **.** Each cross represents a single population.(TIFF)

Table S1 **Population genetic summaries of rarified allelic richness (Ar) and genetic diversity (** ***H_S_*** **).**
(DOC)

Table S2
**Posterior probabilities models excluding one or the other connectivity score.** Our most probable model (bolded) a) included the composite variable 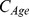 when the composite variable of 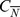 was excluded, while b) the exclusion of the 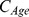 resulted in the null model having the highest posterior probability.(DOC)

Table S3
**Posterior estimates of regression parameters for the model with the highest posterior probability when either (a)** 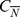 **or (b)** 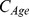 **variable is excluded from the model comparisons.**
(DOC)
