# Supplementary figures and images for: Determinants of Genetic Structure in a Nonequilibrium Metapopulation of the Plant Silene latifolia
Source: PLoS One. 2014 Sep 8;9(9):e104575. doi: 10.1371/journal.pone.0104575 (PMC4157773; doi:10.1371/journal.pone.0104575)

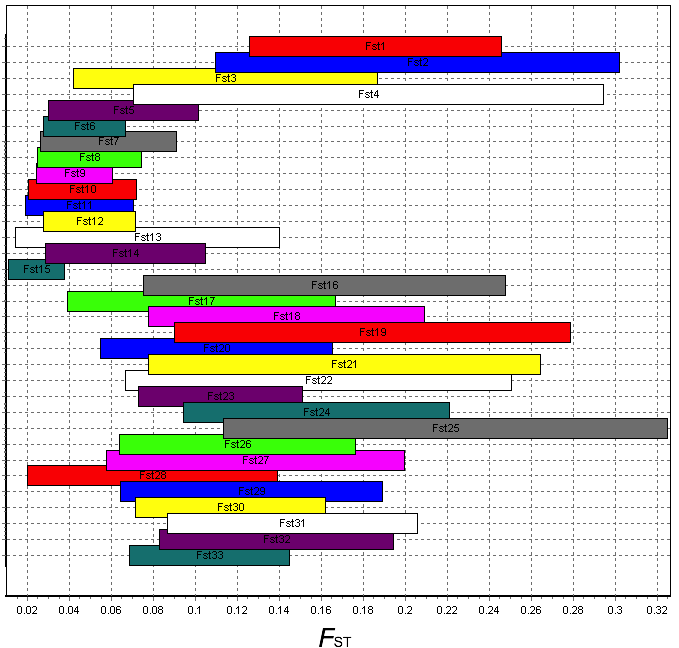

Supplement: Figure S1 — Posterior 95% HPDI estimates of individual population's F -model based estimate of F ST. (TIFF) [file pone.0104575.s001.tiff]

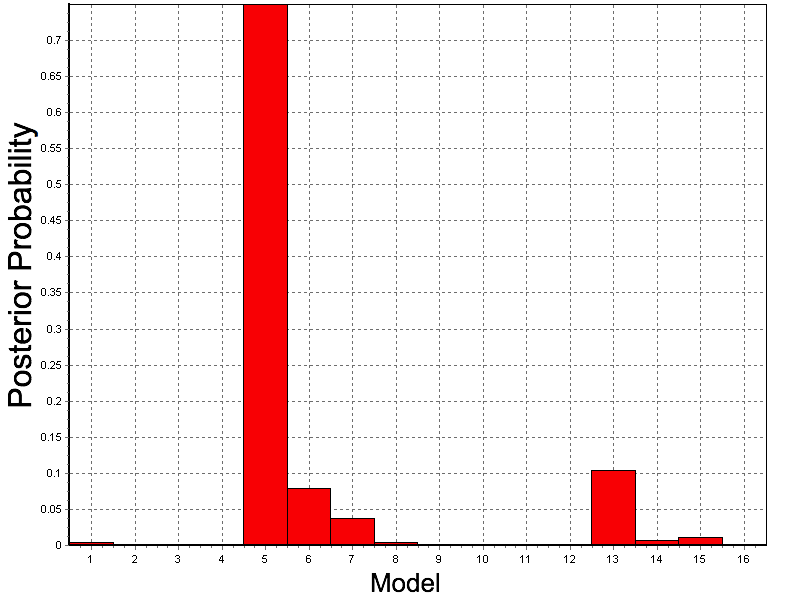

Supplement: Figure S2 — Posterior model probabilities for GESTE run including all four factors. (TIFF) [file pone.0104575.s002.tiff]

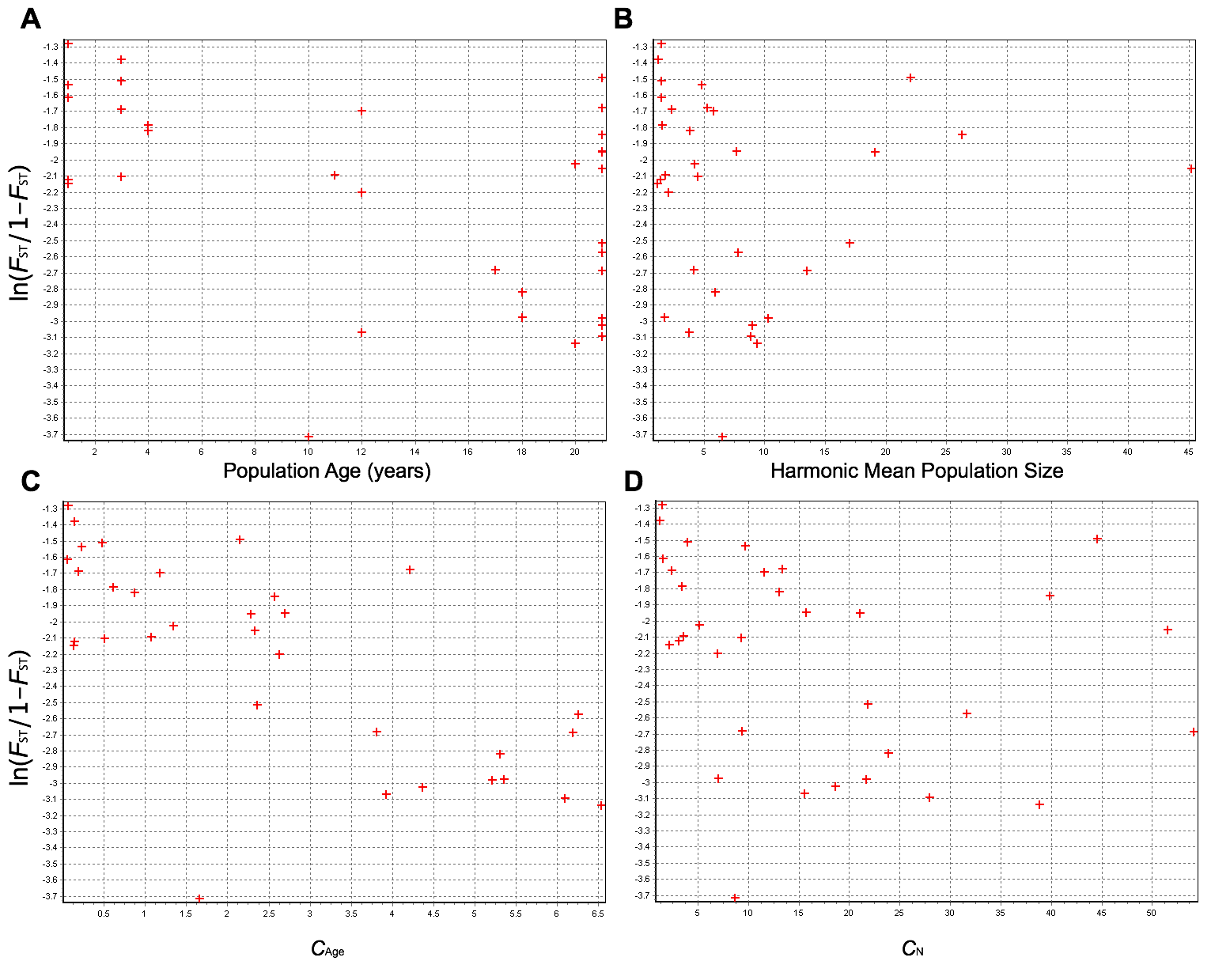

Supplement: Figure S3 — Plots of the GESTE estimated ln( F ST/1- F ST) against (A) population age, (B) Population Size, (C) , and (D) . Each cross represents a single population. (TIFF) [file pone.0104575.s003.tiff]
