# Supplementary material for: Determinants of Genetic Structure in a Nonequilibrium Metapopulation of the Plant Silene latifolia
Source: PLoS One. 2014 Sep 8;9(9):e104575. doi: 10.1371/journal.pone.0104575 (PMC4157773; doi:10.1371/journal.pone.0104575)
Supplement: Table S1 — Population genetic summaries of rarified allelic richness (Ar) and genetic diversity ( HS ). (DOC) [file pone.0104575.s004.doc]

|  | **Diversity Statistics** | | |
| --- | --- | --- | --- |
| **Population ID** | **Sample Size** | **Ar** | ***H*_s_** |
| Population 1 | 24 | 2.345 | 0.623 |
| Population 2 | 12 | 1.980 | 0.457 |
| Population 3 | 7 | 2.116 | 0.525 |
| Population 4 | 5 | 1.886 | 0.422 |
| Population 5 | 19 | 2.245 | 0.552 |
| Population 6 | 50 | 2.127 | 0.492 |
| Population 7 | 22 | 2.107 | 0.519 |
| Population 8 | 24 | 2.277 | 0.562 |
| Population 9 | 39 | 2.255 | 0.542 |
| Population 10 | 21 | 2.261 | 0.561 |
| Population 11 | 18 | 2.372 | 0.587 |
| Population 12 | 47 | 2.097 | 0.485 |
| Population 13 | 8 | 1.890 | 0.44 |
| Population 14 | 20 | 2.122 | 0.511 |
| Population 15 | 44 | 2.259 | 0.537 |
| Population 16 | 11 | 1.746 | 0.355 |
| Population 17 | 8 | 2.010 | 0.456 |
| Population 18 | 19 | 1.880 | 0.41 |
| Population 19 | 10 | 1.948 | 0.465 |
| Population 20 | 16 | 2.244 | 0.554 |
| Population 21 | 10 | 1.995 | 0.49 |
| Population 22 | 6 | 2.044 | 0.518 |
| Population 23 | 36 | 2.261 | 0.589 |
| Population 24 | 27 | 2.010 | 0.466 |
| Population 25 | 8 | 1.876 | 0.437 |
| Population 26 | 21 | 2.018 | 0.459 |
| Population 27 | 8 | 2.318 | 0.629 |
| Population 28 | 4 | 2.318 | 0.583 |
| Population 29 | 15 | 2.241 | 0.556 |
| Population 30 | 44 | 2.216 | 0.574 |
| Population 31 | 24 | 2.182 | 0.543 |
| Population 32 | 29 | 2.071 | 0.506 |
| Population 33 | 44 | 2.227 | 0.529 |
