# Supplementary material for: Determinants of Genetic Structure in a Nonequilibrium Metapopulation of the Plant Silene latifolia
Source: PLoS One. 2014 Sep 8;9(9):e104575. doi: 10.1371/journal.pone.0104575 (PMC4157773; doi:10.1371/journal.pone.0104575)
Supplement: Table S2 — Posterior probabilities models excluding one or the other connectivity score. Our most probable model (bolded) a) included the composite variable when the composite variable of was excluded, while b) the exclusion of the resulted in the null model having the highest posterior probability. (DOC) [file pone.0104575.s005.doc]

| Model | Pr | Factors included |
| --- | --- | --- |
| (a) |  |  |
| **5** | **0.86** | $C_{\overline{N}}$ |
| 6 | 0.08 | $C_{Age},\text{Population Age}$ |
| 7 | 0.04 | $C_{Age},\overline{N}$ |
| 1 | 0.01 | $\text{Null}$ |
| 8 | 0.00 | $C_{Age},\overline{N},\text{Population Age}$ |
| 2 | 0.00 | $\text{Population Age}$ |
| 3 | 0.00 | $\overline{N}$ |
| 4 | 0.00 | $\overline{N},\text{Population Age}$ |
| (b) |  |  |
| **1** | **0.58** | $\text{Null}$ |
| 2 | 0.29 | $\text{Population Age}$ |
| 4 | 0.04 | $\overline{N},\text{Population Age}$ |
| 5 | 0.04 | $C_{\overline{N}}$ |
| 3 | 0.02 | $\overline{N}$ |
| 6 | 0.02 | $C_{\overline{N}},\text{Population Age}$ |
| 7 | 0.01 | $C_{\overline{N}},\overline{N}$ |
| 8 | 0.00 | $C_{\overline{N}},\overline{N},\text{Population Age}$ |
