# Supplementary material for: Determinants of Genetic Structure in a Nonequilibrium Metapopulation of the Plant Silene latifolia
Source: PLoS One. 2014 Sep 8;9(9):e104575. doi: 10.1371/journal.pone.0104575 (PMC4157773; doi:10.1371/journal.pone.0104575)
Supplement: Table S3 — Posterior estimates of regression parameters for the model with the highest posterior probability when either (a) or (b) variable is excluded from the model comparisons. (DOC) [file pone.0104575.s006.doc]

| Regression coefficient | Factor | Mean | Mode | 95% HPDI |
| --- | --- | --- | --- | --- |
| (a) |  |  |  |  |
| $\alpha_{0}$ | Constant | -2.26 | -2.26 | [-2.51; -2.00] |
| $\alpha_{3}$ | $C_{Age}$ | -0.435 | -0.445 | [-0.687; -0.193] |
| $\sigma^{2}$ | - | 0.369 | 0.322 | [0.167; 0.614] |
| (b) |  |  |  |  |
| $\alpha_{0}$ | Constant | -2.25 | -2.25 | [-2.53; -1.96] |
| $\sigma^{2}$ | - | 0.534 | 0.462 | [0.256; 0.874] |
